# IPVAW male perpetrators convicted in Spain: a typology and characterization based on latent class analysis

**DOI:** 10.3389/fpsyg.2024.1353809

**Published:** 2024-03-04

**Authors:** Iria de la Osa-Subtil, Andrés Arias Astray, Pedro V. Mateo Fernandez, María J. de Dios-Duarte

**Affiliations:** ^1^Department of Medicine, Faculty of Biomedical Sciences and Health, European University of Madrid, Madrid, Spain; ^2^Faculty of Psychology, Complutense University of Madrid, Madrid, Spain; ^3^Complutense University of Madrid, Knowledge Technology Institute, Madrid, Spain; ^4^Department of Social Work and Social Services, Faculty of Social Work, Complutense University of Madrid, Madrid, Spain; ^5^Department of Psychology, Faculty of Biomedical Sciences and Health, European University of Madrid, Madrid, Spain; ^6^Faculty of Nursing, University of Valladolid, Valladolid, Spain

**Keywords:** intimate partner violence against women, men, latent class analysis, typologies, classification

## Abstract

**Introduction:**

Men who assault their partners present deficits in the social skills necessary for adequate interpersonal interaction. Not all of them have the same difficulties, thus they do not constitute a homogeneous group. Various studies have proposed different typologies of abusers based on their sociodemographic characteristics, criminal history, intensity and extent of violent or psychopathological traits. The majority of these investigations have been conducted in community samples, prompting the question of their validity in samples of men convicted of gender violence. The aim of this study was to establish a typology of men convicted in Spain for a gender violences crimes.

**Methodology:**

A total of 365 men participated and were subdivided into three classes of abusers based on their childhood, family experiences with violence, criminal history, sexist attitudes and attitudes toward violence, intensity and type of violence, psychopathological state and attachment style.

**Results:**

Coinciding with the results found in other research, 30% of the participants were classified as generally violent. They engaged in severe forms of physical, psychological and sexual violence and were more likely to do so than the rest. Additionally, they are more likely to present psychopathological problems and an antisocial character. Twenty-one percent were classified as dysphoric/borderline. They are characterized by minor forms of psychological violence, borderline or depressive symptomatology and an anxious attachment style. The remaining 49% were classified as familial or normalized abusers. This group exhibits moderate attitudes toward violence and sexism, resulting in less psychological and physical aggression. They do not present psychopathological problems and are likely to present a secure attachment style.

**Discussion:**

It is argued that determining the psychological characteristics of each type of abuser would contribute to improving and adapting intervention protocols in Spain, leading to a significant improvement in the current issue of abuse.

## 1 Introduction

Intimate partner violence against women (IPVAW) is a complex phenomenon consisting of multiple factors. It involves structural, community, interconnected and individual variables (Heise, 1998). In relation to the latter, it is known that gender aggressors have fewer psychological and social resources and have more difficulties in carrying out adequate interpersonal interaction than non-violent men (Holtzworth-Munroe et al., 1997). These difficulties can be subdivided into cognitive, behavioral-relational and emotional (Redondo and Graña, 2012). With respect to the cognitive area, aggressors tend to minimize the consequences of the use of violent behavior, to externally attribute responsibility for their behavior, and to maintain beliefs and attitudes based on traditional gender roles (Fernández-Montalvo and Echeburúa, 1997; Sonkin and Dutton, 2003). At the relational behavioral level, deficits in social and communication skills, interaction style, need for control and dissatisfaction in the relationship are also usually involved in the explanation of violence against partners (Cantos et al., 1994; Jacobson et al., 1994; Anglin and Holtzworth-Munroe, 1997). Finally, among the psychopathological-affective variables, inadequate emotional regulation of anger or jealousy, high impulsivity, anxious-depressive symptomatology, attachment style, and presence of antisocial and borderline personality traits are usually observed (Murphy et al., 1993; Pan et al., 1994; Holtzworth-Munroe et al., 1997; Andrews et al., 2000; White and Gondolf, 2000; Sommer et al., 2017).

In addition to the above, learning history and life trajectory have also been shown to be relevant in explaining IPVAW. Some models, such as that of Cascardi and Jouriles (2018), indicate that, despite the inability to establish a causal relationship in the use of violence, experiencing violence in childhood poses a risk factor for using violence in adulthood. The study of the presence, absence and/or combination of these risk variables has revealed the heterogeneity of gender abusers. This has led to various efforts to establish an adequate classification of these. In addition, the etiological mechanisms of IPVAW have been studied in depth (Capaldi and Kim, 2007). One of the most well-known and widely replicated typologies of IPVAW perpetrators is the one proposed by Holtzworth-Munroe and Stuart (1994).

These authors distinguish three subtypes of offenders: family-only (OF), generally violent (GV), and borderline/dysphoric (BD). They classify offenders according to the intensity and frequency of the violence perpetrated, both within and outside the family setting and according to their psychopathological characteristics. OF aggressors (50%) perpetrate violence in the family setting, present a low level of violence, a normal psychological profile and good social adaptation. GV perpetrators (25%) are violent both with their partners and in other contexts, present a severe level of violence, antisocial traits and difficulties in social adaptation. Finally, BD offenders (25%) may present borderline or dysphoric symptomatology, such as emotional instability, impulsivity, dependence, fear of abandonment or insecure attachment. In addition, BD offenders present a moderate level of violence use accompanied by a dysfunctional psychological profile and variable social adjustment. Based on these results, Holtzworth-Munroe (2000) conducted another investigation in which they identified a fourth group of abusers, in which low-level antisocial characteristics were prominent (LLA; 33%). This group was between OF (36%) and GV (16%) offenders.

In this taxonomic effort there are numerous recent studies that, also based on risk variables or on the level of the risk of violence have established different typologies that include two (Loinaz et al., 2010; Loinaz, 2014; Teva et al., 2023), three (Babcock et al., 2000; Langhinrichsen-Rohling et al., 2000; Waltz et al., 2000; Stoops et al., 2010; Graña et al., 2014), four (Eckhardt et al., 2008; Thijssen and de Ruiter, 2011; Weber and Bouman, 2020; González-Álvarez et al., 2022), or up to five types of abusers (Chiffriller and Hennessy, 2010). However, there seems to be a high degree of consensus on the existence of the three types of violent men initially noted, especially with regard to GV and FO abusers (Weber et al., 2019), there being, generally speaking, a temporal stability of abusers in their corresponding classification (Cavanaugh and Gelles, 2005).

Given the relational nature of IPVAW, some of the classifications have studied its different types taking into account the attachment style. Understanding the insecure attachment style as a risk variable (De la Osa et al., 2022), this attachment style has been related to the use of aggression in the couple (Dutton, 1995; Babcock et al., 2000; Oka et al., 2014; Barbaro et al., 2019). In contrast, a secure attachment style has been linked to the use of prosocial behaviors (Mikulincer and Shaver, 2011). According to Holtzworth-Munroe and Stuart's (1994) model, the GV group tends to maintain an avoidant attachment style, whereas the BD tends to present a preoccupied or ambivalent attachment style and the OF a secure or possibly ambivalent attachment style. Similarly, Chiffriller and Hennessy (2010) have found that BD offenders show a greater preoccupied attachment than those in the GV group, also that they are more fearful than GV and OF. Regarding secure and avoidant attachment style, these authors found no differences.

In a study in which similar categories were posed, Waltz et al. (2000) found significant differences between attachment styles and types of abusers. GV abusers showed more avoidant and less anxious attachment patterns than DFs. The latter presented higher levels of anxious-ambivalent attachment than GVs. On the other hand, Mauricio and Lopez (2009) found three types of abusers that they categorized according to the level of dangerousness, from less to more violent. Their results showed a positive correlation between certain attachment styles and belonging to more violent types. Specifically, it was found that an increase in borderline personality disorder, anxious attachment, and avoidant attachment scores was associated with a greater likelihood of belonging to the most violent class.

In none of the previous studies was attachment style included as a classification variable, but rather differences were assessed once the groups were established.

Several recommendations have been proposed for establishing classifications with IPVAW offenders. Firstly, typology studies have often been developed with community samples, but it is recommended that they be conducted with specific samples, such as men convicted by a court (Capaldi and Kim, 2007). Secondly, self-reported measures are more accurate for offender classification than other types of measures (Weber et al., 2019). Finally, as a statistical tool for establishing typologies in this area, the use of Latent Class Analysis (LCA) is recommended.

Considering all of the above, the general objective of this study is to obtain a classification of men convicted of IPVAW in Spain according to the type and intensity of the aggression, their past experiences of violence, their perception of the use of violence, and their psychopathological and attachment style characteristics, using LCA, as this is the analytical technique of choice.

It is expected to identify different groups with characteristics similar to those found by Holtzworth-Munroe and Stuart (1994). Also, it is expected to know the consistency and usefulness of the classification, checking whether the groups identified were related to external variables relevant to the phenomenon under study.

## 2 Methodology

### 2.1 Participants

The sample consisted of 365 men ranging in age from 19 to 80 years, the mean age was 39.6 years (SD = 11.4). Most of the men were European (*n* = 241, 76.1%). Of these, 217 were Spaniards (68.5%). 17% percent were Latin American (*n* = 54), 6% from Africa (*n* = 19) and the remaining 0.9% from Asia (*n* = 3). Of the men, 59.7% (*n* = 157) were middle class, 24.3% (*n* = 64) lower class and 16% (n = 42) upper class. The educational level of the participants was unevenly distributed. 51.7% (*n* = 155) had a high school level of education, 28.2% (*n* = 83) had completed primary education, 13.3% (*n* = 39) had completed university studies and 5.8% (*n* = 17) had no education at all.

### 2.2 Design and procedure

The participants in this study had been convicted of a crime of violence against their partner and were in a situation of substitution of sentence conditioned to a psychological treatment program according to Article 35 of Section IV of the Spanish Organic Law 2004 on Gender Violence, which indicates that men convicted of this type of crime must mandatorily attend a specific program of re-education and psychological treatment. Following a court order, participants are summoned to the corresponding CIS and in an individual interview, CIS professionals carry out the screening of criteria and the allocation of a treatment group to the timetable.

Data were obtained through an evaluation protocol in Spanish applied in the first session of the treatment program, which lasted approximately 2 h in total. Prior to responding the protocol, participants received information about the study’s objectives and their involvement in it. Clear instructions on completing the questionnaires were provided, according to the ethical considerations on participation in research proposed by the APA (American Psychological Association) in “The ethical principles of psychologists and code of conduct,” the Ethical Principles for Human Research of the Declaration of Helsinki and the Principles of the Deontological Code of the Psychologist (section IV) of the General Council of Psychology in Spain. All participants voluntarily and altruistically signed the informed consent form to participate in this research.

The sampling approach was non-probabilistic and of convenience and the design was observational, analytical, prospective and cross-sectional. The exclusion criteria established in this study were: having served the sentence, being a minor, and not knowing how to read or not understanding Spanish correctly. It was no necessary to exclude any participant. In order to carry out this study, favorable reports were obtained from the deontological committee of the Faculty of Psychology of the Complutense University of Madrid and the authorization of the Secretariat of Penitentiary Institutions of the Spanish Government, before starting the research.

### 2.3 Instruments

#### 2.3.1 Sociodemographic questionnaire

A questionnaire was created *ad hoc* to assess the sociodemographic and personal characteristics of the participants, including age, nationality and level of education. Additionally, questions were posed regarding criminal history, perceived childhood abuse by parents and other caregivers, as well as experiences and observations of violence by their father toward their mother.

#### 2.3.2 Conflict tactics scale (CTS-2; Straus et al., 1996)

This scale measures the frequency of the use of psychological and physical aggression, as well as the use of negotiation strategies in couple relationships. Its psychometric properties were reviewed in the abuser population (Loinaz et al., 2012). This scale consists of 78 items (39 for each partner). It is a 7-point Likert-type scale, ranging from 0 to 6 where 0 equals never and 6 equals more than 20 times. It allows scores to be obtained on a ratio scale. It contains 10 scales of which 6 were used for this study, presenting the following reliability coefficients for this population: minor (*ω* = 0.827) and severe (*ω* = 0.878) physical violence, minor (*ω* = 0.772) and severe (*ω* = 0.639) psychological aggression, and minor (*ω* = 0.644) and severe (*ω* = 0.845) sexual coercion.

#### 2.3.3 Structured clinical interview for DSM-IV Axis II disorders (SCID-II; First et al., 1999)

It consists of 119 items that reflect the presence or absence of different personality disorders. In this study we used the 30 items that assess the presence of borderline (BPD) or antisocial personality disorder (ASPD). The items have 3 response options (never, sometimes and always or almost always), obtaining the following reliability coefficients: *ω* = 0.886 for the BPD scale and *ω* = 0.894 for the ASPD scale.

#### 2.3.4 State–trait anger expression inventory (STAXI-2; Spielberger, 1988)

This inventory provides a measure of trait anger through two subscales (anger temperament and anger reaction) and of anger state through 3 subscales (feeling, physical expression and verbal expression). An index of anger expression can also be obtained through 4 subscales (external expression of anger, internal expression of anger, external control of anger and internal control of anger). It consists of 49 items with a 4-point scale including the responses “no,” “not at all,” “somewhat,” “moderately” and “very much.” Reliability indices in this sample were: *ω* = 0.944 for the anger-state anger scale, *ω* =0.896 for anger-trait, and *ω* =0.856 for anger expression.

#### 2.3.5 Plutchik impulsivity scale (EI-Is; Plutchik and Van Praag, 1989)

It consists of 15 items with four response options (never, sometimes, often and almost always) that indicate the tendency to act impulsively through its four subscales (ability to plan; control of emotional states; control of eating behaviors, spending money or maintaining sexual relationships and control of other behaviors). The reliability coefficient of the total scale was *ω* = 0.781.

#### 2.3.6 Brief symptom inventory (BSI; Derogatis and Melisaratos, 1983)

This is a dimensional inventory adapted to Spanish by Aragón et al. (2000) that evaluates symptomatology in nine scales, of which two were used in this study: anxiety and depression. The total scale has 53 items with Likert-type response alternatives from 0 to 4. In this population, the reliability indices were optimal (*ω*_anxiety_ = 0.867, *ω*_depression_ = 0.910).

#### 2.3.7 Inventory of distorted thoughts about women and the use of violence-revised (IPDMUV-R, Echeburua et al., 2016)

It is an instrument that assesses the cognitive biases against the partner presented by violent men. This version is derived from the IPDMUV (Spanish acronym) (Fernández-Montalvo and Echeburúa, 1997). It consists of 21 binary items that form a single scale that allows the identification of irrational beliefs in the aggressor related to gender roles and the supposed inferiority of women with respect to men, as well as the use of violence as an acceptable way to resolve conflicts. The reliability coefficient of the total scale was *ω* = 0.777.

#### 2.3.8 Dominating and jealous tactics scale (Kasian and Painter, 1992)

It is a scale composed of 22 items, 11 of which were obtained from the Psychological Maltreatment of Women Inventory by Tolman (1989). Its objective is to assess various forms of emotional aggression in intimate relationships with 5 response alternatives ranging from 1 (never) to 5 (very often) estimating the frequency with which dominating and jealous tactics are used by the respondent and her partner. In our study we included the 7 items assessing dominating tactics and the 4 items assessing jealous tactics on the part of the aggressor. In this sample, the coefficient ω was 0.886.

#### 2.3.9 Justification of verbal/coercive tactics scale (JVCT; Smith et al., 2001)

It has 26 items (13 for men and 13 for women) with 5 response alternatives ranging from 1 (never justified) to 5 (justified on many occasions). In this research, the scale was used for men to women obtaining a *ω* = 0.846.

#### 2.3.10 Attitudes toward interpersonal violence (AIV; Riggs and O'Leary, 1996)

It assesses beliefs associated with justifying physical aggressions (pushing, slapping and hitting) between men and women through 6 items with 5 response alternatives ranging from 1 (never) to 5 (very often). The male-on-female violence attitudes scale was chosen for this study (*ω* = 0.809).

#### 2.3.11 Adult attachment questionnaire (AAC; Melero and Cantero, 2008)

The Melero and Cantero Adult Attachment Questionnaire consists of 40 items on a Likert-type scale (1–6). It evaluates different dimensions of attachment in adults. Its items form part of a latent structure of 4 factors which, grouped together, give rise to the attachment styles theorized, both bidimensional (secure and insecure) and categorical (secure, preoccupied, fearful-hostile, avoidant). The reliability indices for this sample were: Scale 1: low self-esteem, need for approval and fear of rejection (*ω* = 0.851); Scale 2: hostile conflict resolution, resentment and possessiveness (*ω* = 0.818); Scale 3: expression of feelings and comfort with relationships (*ω* = 0.787); Scale 4: emotional self-sufficiency and discomfort with intimacy (*ω* = 0.653).

### 2.4 Data analysis

The R program (RStudio, 4.2.3) was used to perform all the analyses. First, a descriptive analysis of the variables and their reliability indices was conducted. Second, a latent profile analysis was conducted to determine the attachment styles of the adult attachment scale. Third, Latent Class Analysis (LCA) was used to identify abuser typologies. LCA attempts to identify latent variables through the relationships between observed variables and to obtain patterns underlying the data, as opposed to other clustering techniques with which similarity or relatedness between observed data is obtained. Together with k-means cluster analysis, LCA has established itself as a methodologically sound technique in IPVAW offender classification (Alexander and Johnson, 2023). But LCA has certain advantages over k-means (Magidson and Vermunt, 2002) because it is based on a probabilistic model that allows cases to be assigned to clusters more accurately and error rates to be estimated. It provides objective fit criteria to determine the number of clusters. It does not require standardization of the variables, since the solution is invariant to linear transformations and, finally, it allows the use of more flexible and complex models that include variables of different natures. It also incorporates covariates that make the description of the clusters possible, since it does not require the assumption of continuous data. Multinomial variables, that are frequently used in this type of classification, can be included (McKay et al., 2022). This technique uses indicator variables (categorical) to identify latent and unobservable patterns of homogeneous groups within a more general group, finally obtaining the probability of class membership for each individual (Muthén and Muthén, 2002).

The variables included were dichotomized in terms of presence/absence according to the cut-off points stipulated by the authors or frequency (in terms of presence and absence). To choose the best of the models, statistical criteria were evaluated, but also theoretical criteria, because the model must be able to be interpreted and make theoretical sense (Muthén and Muthén, 2002; Nylund et al., 2007). The theoretical foundation of the typologies was that proposed by Holtzworth-Munroe and Stuart (1994). Model fitting was based on log likelihood descent, Akaike information criterion (AIC; Akaike, 1987), Conditional Akaike information criterion (CAIC, Saefken et al., 2021), Bayesian information criterion (BIC; Schwarz, 1978) and its variation adjusted for sample size (SABIC, Schwarz, 1978; Sclove, 1987). Although it was not a criterion for comparing models, entropy greater than 0.8 was sought (Muthén, 2008), the smallest class size was taken into account (Chen et al., 2017), and the tendency to overfit models with having many parameters was assessed (Sinha et al., 2021). After model choice, classes were assigned to each case and the probabilities of belonging to each class were obtained. Finally, the association between the assigned class membership and other variables was investigated. Its relationship with dominant and jealous tactics and the use of coercive and verbal tactics was reviewed by fitting a multiple linear regression for each variable.

## 3 Results

### 3.1 Distribution of types of aggression and risk variables

In the context of this study (see Table 1), more than half of the participants had experienced child abuse by a relevant figure. Most of them had not experienced IPVAW between their parents nor possessed a criminal record. Minor psychological violence was the most frequent form of aggression in this population, followed by minor physical violence and severe psychological violence. Almost half had borderline personality disorder and in a few cases other psychopathological problems were present. The most common type of adult attachment was secure, followed by the preoccupied attachment style. Most of the participants justified the use of coercive, verbal, dominating and jealous tactics.

**Table 1:** Characteristics of the sample of men convicted of IPVAW.

	*N* (%)
Sociodemographic groups	
European	241 (76.1%)
Spanish	217 (68.5%)
Latin Americans	54 (17%)
Africans	19 (6%)
Asians	3 (0.9%)
Socioeconomic level	
Low	64 (24.3%)
Medium	157 (59.7%)
High	42 (16%)
Educational level	
No education	17 (5.8%)
Primary	83 (28.2%)
Secondary	155 (51.7%)
University students	39 (13.3%)
Experiences of violence	
Criminal records	129 (35.3%)
Childhood abuse	190 (52.1%)
IPVAW in family of origin	80 (21.9%)
Aggression	
Severe physical	76 (20.8%)
Minor physical	131 (35.9%)
Severe psychological	109 (29.9%)
Minor psychological	224 (61.4%)
Sexual coercion	51 (13.9%)
Psychopathology and emotional regulation	
Presence of BPD	141 (39.6%)
Presence of ASPD	56 (15.7%)
High trait anger	22 (6%)
High state anger	159 (43.6%)
High impulsivity	63 (17.3%)
Severe anxious symptomatology	32 (8.8%)
Severe depressive symptomatology	49 (13.4%)
Adult attachment	
Preoccupied	150 (41.1%)
Secure	167 (45.8%)
Avoidant	21 (5.8%)
Fearful-Hostile	27 (7.4%)
Attitudes toward violence	
Justification for the use of interpersonal violence.	33 (9.0%)
Justification for the use of verbal and coercive tactics	344 (94.2%)
Justification for the use of dominant and jealous tactics.	250 (68.5%)

### 3.2 Latent classes of IPVAW offenders

Three homogeneous groups of abusers were found. Several latent class models with 2, 3, 4, 4, 5 and 6 classes were estimated to subsequently choose the one that presented the best fit in statistical terms and the best construct validity. As shown in Table 2, the BIC suggested a three-class model. On the other hand, the rest of the fit criteria did not indicate a specific model. It is usual for the fit indicators to show inconsistent results, with the BIC being the most reliable fit statistic for the LCA (Nylund-Gibson and Choi, 2018). In addition, the 3-class model provides adequate entropy (>0.80) and percentage of subjects. Integrating statistical fit indicators and theoretical and interpretability criteria, a 3-class model was determined as the most suitable one. The classes are described below in terms of low, moderate and high probability, taking into account the indicator values and their comparison with the rest of the classes.

**Table 2:** Comparison of model fit parameters according to different class solutions.

Numbers of class	LogLik	BIC	SABIC	AIC	CAIC	Entropy	Smallest class size (%)
2	−3,229	6,713	6,576	6,545	6,756	0.836	40%
3	−3,159	6,701	6,495	6,448	6,767	0.810	21%
4	−3,105	6,722	6,446	6,383	6,809	0.794	13%
5	−3,074	6,807	6,444	6,365	6,899	0.801	7.5%
6	−3,046	6,900	6,449	6,389	6,995	0.824	7.3%

LogLik, log likelihood; BIC, Bayesian information criterion; SABIC, sample-adjusted Bayesian information criterion; AIC, Akaike information criterion, CAIC, Conditional Akaike information criterion.

Class 1 (21%; M_age_ = 41.6; 16.4% Spanish, 2.2% Latin American, 1.3% European, 1.3% African, 0% Asian). These individuals were highly likely to have a criminal record (44%), to have been abused in childhood (60%) and to have witnessed violence from their father toward their mother (27%). However, they were less likely to justify the use of physical interpersonal violence than the others (4.6%) and had less gender bias (8%). In terms of aggression, they were highly likely to use minor psychological aggression (52%) and on a moderate to severe level (23%). However, they hardly made use of minor (6.8%) or severe (0%) physical violence or sexual coercion (2%). This group is the one with the greatest psychopathological problems, having a high probability of presenting BPD (69%) and high levels of state anger (68%) and depression (36%). With moderate probability they may present ASPD (15%), high trait anger (27%), impulsivity (10%) and anxiety (18%). This group is characterized by a high probability of presenting a preoccupied attachment style (68%).

Class 2 (49%; M_age_ = 34.9; 33.1% Spanish, 9.1% Latin American, 4.7% European, 2.2% African, 0.6% Asian). These individuals were less likely than the rest to have had a criminal record (28%), to have been abused in childhood (44%), or to have experienced violence from their father toward their mother (13%). They were also moderately likely to justify the use of interpersonal violence (8%) and to hold gender biases (12%). Regarding the intensity and type of aggression, it was highly likely they used minor psychological violence (42.4%) and with less likely they chose minor (19%) and severe physical aggression (8.2%) and severe psychological aggression (4.6%) or sexual coercion (4%). In the psychopathological sphere, they have a very low probability of having psychopathological problems such as BPD (8%), TAP (2%), trait anger (4%) or state anger (3%). In addition, they do not present problems of impulsivity, anxiety and depression (<1%). Regarding attachment style, they present a high probability of maintaining a secure attachment style (65%).

Class 3 (30%; M_age_ = 41.5; 18.9% Spanish, 5.7% Latin American, 1.6% European, 2.5% African, 0.3% Asian). People in this group are the most likely to have a criminal record (44%), to have suffered abuse in childhood (60%), and to have had IPVAW experiences in their family of origin (33%). They also justify the use of violence more than the others (13.6%) and are more likely than the rest to have sexist biases (19%). This group is the one most likely to assault in all its forms, severe physical (55%) and psychological aggression (76%), physical (83%) and minor psychological aggression (99%) and sexual coercion (31%). Their psychopathology is manifested with a high probability of having borderline personality disorder (70.3%) and antisocial personality disorder (37%) and the highest level of trait anger (30%). It is moderately likely that they present high levels of state anger (47%), impulsivity (14%), anxiety (16%) and depression (19%). Their attachment style is predominantly preoccupied (48%).

### 3.3 Effect of class-membership on the use of coercive and dominant tactics

The regression results (see Table 3) indicate that the different classes significantly predict the use of violent tactics, with class 3 being the one that resorts to them the most. First, in the model whose dependent variable is dominating and jealous tactics, the estimated coefficients indicate that, holding all other variables constant, class 3 (*M* = 17.5) uses 4 points on average more of these types of tactics than class 2 (*M* = 15.6) and 1.8 points on average more than class 1 (*M* = 13.4). This model explains 6.1% of the variance. Second, when coercive and verbal tactics are set as the dependent variable, the results indicate that, holding all other values constant, class 3 (*M* = 25.1) uses on average 4 points more of these types of tactics than class 1 (*M* = 20.7) and 5 points more on average than class 2 (*M* = 20). In this case, the model explains 9.3% of the variance.

**Table 3:** Linear regression models.

	Coercive and verbal tactics				Dominant and jealous tactics			
	*B*	SE B	*β*	IC	*B*	SE B	*β*	IC
Class 1–3	−4.38	1.26	−0.50^***^	[−0.78, −0.21]	−1.87	0.90	−0.29^**^	[−0.58,-0.01]
Class 2–3	−5.12	1.03	−0.58^***^	[−0.81, −0.35]	−4.02	0.74	−0.63^***^	[−0.86,- 0.40]
Intercept	25.09^***^	0.817	–	–	17.46^***^	0.588	–	–
*R*^2^ adjusted	0.061				0.093			

^*^p < 0.05; ^**^p < 0.01; ^***^p < 0.001.

## 4 Discussion

The present study was designed to identify different types of intimate partner abusers in a sample of men convicted of gender violence. This was done using risk indicators that have been shown to be relevant in other studies. These indicators include some sociodemographic data, experiences with violence, positive attitudes toward violence and sexist attitudes, type and intensity of aggression, psychopathology and attachment styles. The results revealed the existence of three classes of abusers.

### 4.1 GV-class 3

Thirty percent of the participants were in the most violent group. They were highly likely to present psychopathology and to make frequent and intense use of violence (class 3). This clearly corresponds to Holtzworth-Munroe and Stuart’s (1994) GV group characterized by severe and frequent forms of violence against their partners. They are also distinguished by having mental health problems and criminal records. They also correspond to what have been called generalist (Herrero et al., 2016; Teva et al., 2023) or high-risk (Cavanaugh and Gelles, 2005; Graña et al., 2014) aggressors. The results of our study show that this group presents a high probability of having suffered childhood abuse and IPVAW experiences in their family of origin. In addition, they are more likely than the others to justify the use of violence and present sexist biases. A characteristic element of this group is that they are more likely to use all forms of aggression: severe physical and psychological, minor physical and psychological, and sexual coercion. Finally, they present a high probability of having psychopathological problems such as ASPD and BPD symptomatology, predisposition to perceive situations as hostile, impulsivity and high prevalence of preoccupied attachment. Other works have found a similar profile of aggressors who exert violence more severely and frequently, while presenting antisocial personality disorder (Capaldi and Clark, 1998; Andrews et al., 2000; Petersson et al., 2016) and borderline personality disorder (Herrero et al., 2016). Furthermore, in another study it was observed that these men, in comparison to other groups, present psychological distress, attachment insecurity, childhood trauma and poor affect regulation more frequently, presenting the lowest levels of functioning (Brassard et al., 2023).

### 4.2 DF-class 1

The 21% of the sample constituted another group that revealed a similar configuration to the previous group but differed from it in the form and intensity of aggression. Like the GV group, participants in this class were highly likely to have a criminal record, to have been victims of violence in childhood, to have psychopathological problems such as BPD, and a certain tendency to experience anger, impulsivity, severe depression, and preoccupied attachment style. However, they were moderately likely to use minor psychological aggression. In addition, compared to the rest of the classes, they were the least likely to justify the use of violence and sexist beliefs. This group corresponds to Holtzworth-Munroe and Stuart’s (1994) DF classification. It is characterized by having a problematic psychopathological and relational profile and making moderate use of intimate partner violence. However, in our study, this group was not very violent. This statistic is consistent with the observations of Vignola-Lévesque and Léveillée (2022) who identified a group of gender abusers characterized by mild and moderate aggression, with great problems in managing and verbalizing their anger, anxiety and depressive affects, converging in a possible problem of alexithymia. These authors agree with other studies that have found that some intimate partner abusers have difficulty identifying, verbalizing and regulating their hostility and other negative emotions. All of this ultimately results in a variety of violent behaviors (Dutton, 2007; Piquero et al., 2014; Cunha and Goncalves, 2016) that can sometimes be interpreted as inadequate strategies to avoid abandonment (Norlander and Eckhardt, 2005; Di Piazza et al., 2017). In the case of these men, the use of destructive behaviors with their partners may be reflecting an emotional management problem (Porcelli and Mihura, 2010; Hornsveld and Kraaimaat, 2012). On the other hand, personality disorders have been associated with partner aggression (Dutton, 2007; Collison and Lynam, 2021). In these cases, borderline personality seems to play a mediating role between preoccupied attachment style and aggression (Mauricio et al., 2007).

### 4.3 OF-class 2

According to the OF subtype proposed by Holtzworth-Munroe and Stuart (1994), which is considered a normalized subtype (sharing characteristics with non-violent men), in our work we found a last and third large group (41% of the sample) that differed from the rest mainly in psychopathology, attachment style and intensity of aggression. These individuals were least likely to have a criminal history, to have been abused in childhood, or to have witnessed violence from their father toward their mother. It was moderately likely that they used psychological aggression and less likely to use physical aggression. The main difference with the previous group (DF-Class 1) is that they did not present any psychopathological problems, and also maintained a secure attachment profile, although they had a moderate level of state anger. Despite having a lower profile overall, compared to the rest of the groups, these men moderately justified the use of interpersonal violence and maintained gender biases. In line with the existing literature, this group coincides with specialist aggressors (Herrero et al., 2016; Teva et al., 2023) or those with low levels of physical and psychological aggression (Cavanaugh and Gelles, 2005; Graña et al., 2014). It is estimated that most IPVAW perpetrators would be classified in this subtype if samples were recruited from clinical and community samples (Dixon and Browne, 2003). As we found, and as observed in other research, these perpetrators showed low levels of traits related to personality disorders (Petersson and Strand, 2020) and sexual coercion toward their partners (Chiffriller and Hennessy, 2006; Graña et al., 2014). They also presented lower levels of anger than other groups (Johnson et al., 2006; Stoops et al., 2010; Graña et al., 2014), stereotypical male behaviors (Langhinrichsen-Rohling et al., 2000; Lawson et al., 2003) and violent (Petersson et al., 2016) and sexist (Herrero et al., 2016) attitudes. However, with respect to the latter two attitudes, in our classification this group showed an intermediate profile. Nevertheless, attitudes of normalization of violence and victim blaming provided a climate in which the use of violence is more easily allowed (Martín-Fernández et al., 2018). Therefore, in working with this type of men it is very important to keep in mind that a reduction of cognitive distortions in relation to women and violence can generally improve the IPVAW phenomenon (Carbajosa and Boira, 2013; Echeburúa, 2013; Lila et al., 2013).

With respect to the use of coercive, verbal, dominating and jealous tactics, studies suggest that coercive violence is linked to broader patterns of partner domination and control. Thus, violence that occurs as a result of conflict should be distinguished from that which is premeditated (Johnson, 2008; Hardesty et al., 2015). This would explain why in our research the most violent group (GV-Class 3) was also the one that used this type of tactics more frequently compared to the rest. On the other hand, the other two groups, with less deviant profiles, show relatively lower levels of coercive and dominating tactics. The difference between the latter resided in the use of dominating and jealous tactics, as the group corresponding to the FOs presented significantly more control and jealousy problems than those in the DB group.

**Figure 1:**
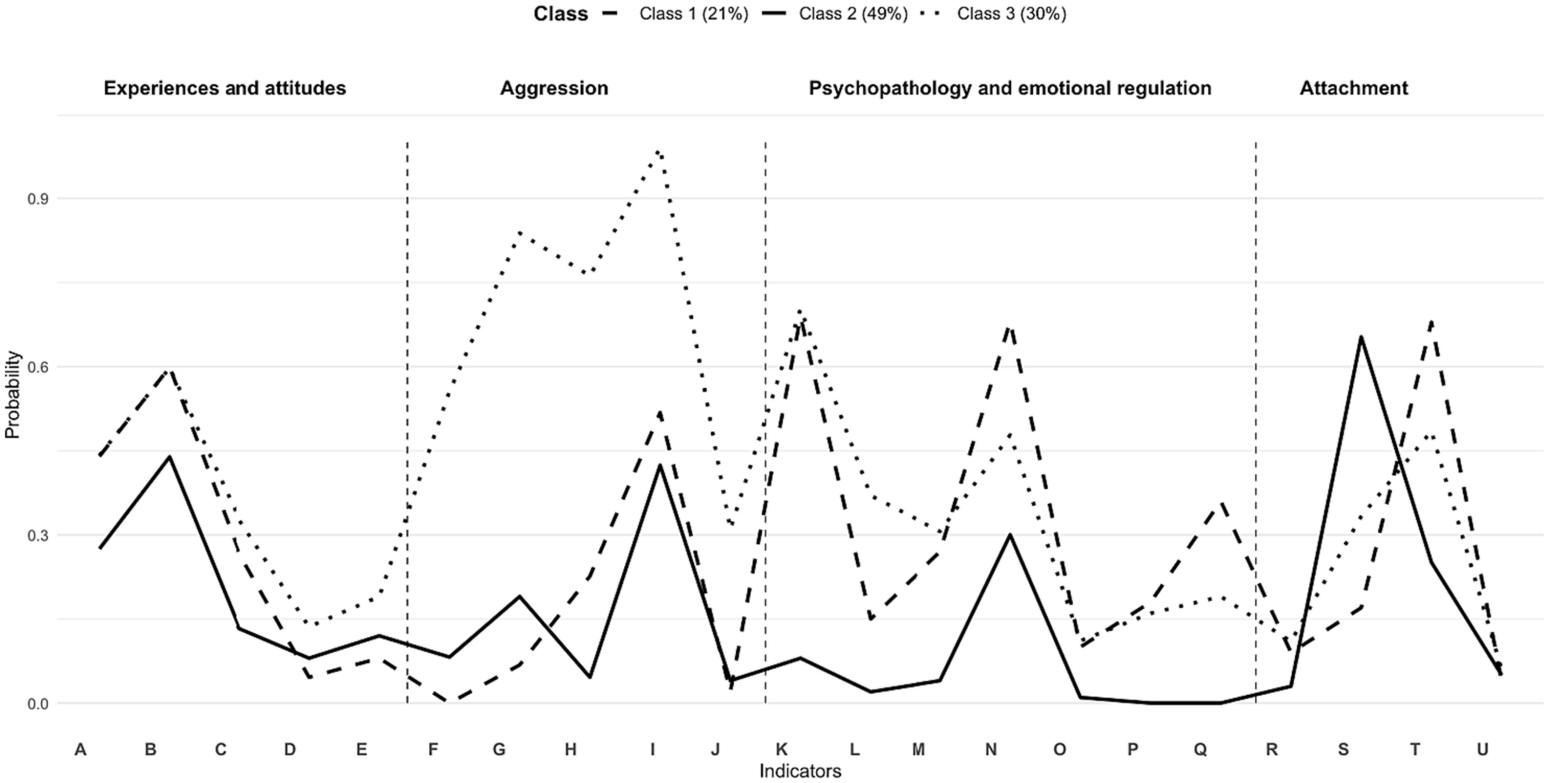
Probability of characteristics and risk factors for each class. A, Criminal history; B, Chilhood abuse; C, Family IPVAW; D, Attitudes toward violence; E, Gender bias; F, Severe physical aggression; G, Minor physical aggression; H, Severe psychological aggression; I, Minor psychological aggression; J, Sexual coercion; K, BDP; L, ASPD; M, Trait anger; N, State anger; O, Impulsivity; P, Anxiety; Q, Depression; R, Fearful; S, Secure; T, Preoccupied; U, Avoidant.

In our study, although differences are observed among the different classes, it seems that all of them have a low to moderate probability and a similar distribution in the presence of criminal history, childhood abuse experiences and having experienced IPVAW from their father toward their mother. With respect to criminal history, it appears that when criminal history is present within the intimate relationship (Campbell et al., 2007) it is a risk factor for re-offending (Piquero et al., 2006). A criminal history was present in 35.3% of our sample, a figure close to that found in other studies with similar populations (Abrunhosa et al., 2020). Regarding the influence of childhood experiences of abuse and violence, the literature suggests that exposure to family violence of origin is a key risk factor for perpetrating IPVAW (Delsol et al., 2003; Godbout et al., 2009; Fulu et al., 2017; Davis et al., 2019; Teva et al., 2020). Our results show that 52.1% of gender batterers have been physically or psychologically abused by a close family member. The latter is a value that is within the range established in the literature regarding perpetrators (Mbilinyi et al., 2012; Lee et al., 2013) and exceeds the rates estimated in general population in Europe (Stoltenborgh et al., 2013, 2015). In contrast, child abuse alone is not a determinant but a risk factor for aggression, particularly in combination with other variables (Figure 1).

Finally, participants’ anger-status levels were high, even in those groups in which they did not have psychopathological problems. This could be indicating that the participants believed that their experience of the situation and evaluation context as unfair and potentially hostile. Possibly, these results are due to a situational state, given that the evaluation occurred in the first session of the treatment group they were obliged to attend. It should be noted that these levels of anger are indicative of the main problem in intervening with this population, which is the defensive attitude with which they begin treatment (Langlands et al., 2009; Lila and Gracia, 2010).

This study has some limitations that should be considered when interpreting the results. First, the sample is composed of men convicted of gender violence who were in the first session of an intervention program as an alternative to imprisonment. This undoubtedly may introduce a selection bias and reduce the representativeness and generalizability of the findings presented here to other populations. Second, the instruments used to assess the variables of interest are participant self-reported measures, which may lead to socially desirable responses. Third, the AAC avoidance scale and the CTS sexual coercion scale showed low reliability ratings and their presence in these participants was low. This could possibly be underrepresenting the importance of these variables in the study. Fourth, the study relies on a single source of information (the subjects themselves) to categorize the participants, which may generate a partial view of reality.

It would be convenient, for future studies, to contrast these data with other sources, such as victims, witnesses or police records, to obtain a more complete and accurate view of the context in which the phenomenon is generated (Hamby, 2017). Additionally, the integration of other dimensions of the ecological model could be further explored, particularly through the lens of gender bias and the construction of masculinity as influenced by context, and their impact on the attitudes and behaviors of various offender types. On one hand, the perceived failure to conform to societal norms of masculinity may be linked to aggression in IPVAW as suggested by Reidy et al. (2014). This could correlate with varying levels of social tolerance and leniency toward IPVAW. In societies marked by violence, such aggression is more prevalent in contexts of isolation, resource scarcity, conservatism, and gender bias (Edwards, 2015; Richardson et al., 2023). On the other hand, recent years have seen the rise of discourses supporting gender equality and opposing sexism, bringing attention to subtler forms of sexism, known as ‘micromachismos’ (Cuenca, 2023). This shift may influence results, as current measurements suggest men do not exhibit gender bias in traditional ways. Therefore, a deeper investigation into this variable could add more nuance to the gender bias factor and enhance the findings. Furthermore, to better understand offender types, future research could compare men convicted of IPVAW who are serving custodial sentences with those serving alternative sentences, anticipating differences within this population. Lastly, considering our study’s findings on attachment style, it would be insightful to determine whether fearful and avoidant attachment styles are less common in this population or if there are more suitable methods of measuring this construct among them.

In conclusion, this research uses indicators that have been shown to be relevant in other similar studies on typologies, such as sociodemographic data, history of violence, attitudes toward violence and sexist attitudes, types and intensity of aggression, psychopathology and attachment styles. These indicators can be used to evaluate the profile and risk of each aggressor, as well as to design strategies and therapeutic objectives appropriate to each case (Babcock et al., 2004). The results of this work reveal the existence of three classes of abusers that are strongly related to the widely known typology of Holtzworth-Munroe and Stuart (1994). In this research, a first class of abusers was found with a low probability of physical aggression, psychopathological problems and a preoccupied attachment style (DF). A second class was also found with a low probability of severe physical and psychological aggression and a secure attachment style (OF). Finally, a third class was found with a high probability of aggression in all its forms and major psychopathological problems and a preoccupied attachment style (GV). These types may have implications for prognosis, treatment and victim protection, given that they respond differently to treatment (Redondo and Graña, 2012). Thus, GV offenders tend to have lower rates of treatment completion and higher rates of recidivism after conviction (Weber et al., 2019). Finally, this study contributes to the advancement of scientific knowledge about the individualities of IPVAW offenders, a complex and multidimensional social phenomenon that requires a comprehensive and multidisciplinary approach (Olver et al., 2011).

## Data availability statement

The raw data supporting the conclusions of this article will be made available by the authors, without undue reservation.

## Ethics statement

The studies involving humans were approved by Deontological Commission of the Faculty of Psychology of the Complutense University of Madrid. The studies were conducted in accordance with the local legislation and institutional requirements. The participants provided their written informed consent to participate in this study.

## Author contributions

IO-S: Conceptualization, Data curation, Formal analysis, Funding acquisition, Investigation, Methodology, Project administration, Resources, Software, Visualization, Writing – original draft, Writing – review & editing. AA: Conceptualization, Funding acquisition, Project administration, Resources, Supervision, Validation, Writing – review & editing, Investigation. PM: Project administration, Resources, Writing – original draft, Data curation. MD-D: Supervision, Validation, Writing – review & editing.
